# Effects of lesions of the subthalamic nucleus/zona incerta area and dorsomedial striatum on attentional set-shifting in the rat

**DOI:** 10.1016/j.neuroscience.2016.08.008

**Published:** 2017-03-14

**Authors:** David S. Tait, Janice M. Phillips, Andrew D. Blackwell, Verity J. Brown

**Affiliations:** School of Psychology and Neuroscience, University of St Andrews, St Andrews, Fife KY16 9JP, UK

**Keywords:** 5CSRTT, five-choice serial reaction time task, 6-OHDA, 6-hydroxydopamine, ANOVA, analysis of variance, BG, basal ganglia, CD, compound discrimination, DA, dopamine, DBS, deep brain stimulation, DLS, dorsolateral striatum, DMS, dorsomedial striatum, DS, dorsal striatum, ED, extradimensional, i.p., intraperitoneal, ID, intradimensional, mPFC, medial prefrontal cortex, NDMA, *N*-methyl-d-aspartate, PD, Parkinson’s disease, PFC, prefrontal cortex, REV, reversal, SD, simple discrimination, SEM, standard error of the mean, STN, subthalamic nucleus, WCST, Wisconsin Card Sorting Test, ZI, zona incerta, basal ganglia, Parkinson’s disease, dopamine, subthalamic nucleus, attentional set-shifting

## Abstract

Patients with Parkinson’s disease (PD) show cognitive impairments, including difficulty in shifting attention between perceptual dimensions of complex stimuli. Inactivation of the subthalamic nucleus (STN) has been shown to be effective in ameliorating the motor abnormalities associated with striatal dopamine (DA) depletion, but it is possible that STN inactivation might result in additional, perhaps attentional, deficits. This study examined the effects of: DA depletion from the dorsomedial striatum (DMS); lesions of the STN area; and the effects of the two lesions together, on the ability to shift attentional set in the rat.

In a single session, rats performed the intradimensional/extradimensional (ID/ED) test of attentional set-shifting. This comprises a series of seven, two-choice discriminations, including acquisitions of novel discriminations in which the relevant stimulus is either in the currently attended dimension (ID) or the currently unattended dimension (ED shift) and reversals (REVs) following each acquisition stage.

Bilateral lesions were made by injection of 6-hydroxydopamine (6-OHDA) into the DMS, resulting in a selective impairment in reversal learning. Large bilateral ibotenic acid lesions centered on the STN resulted in an increase in trials to criterion in the initial stages, but learning rate improved within the session. There was no evidence of a ‘cost’ of set-shifting – the ED stage was completed in fewer trials than the ID stage – and neither was there a cost of reversal learning. Strikingly, combined lesions of both regions did not resemble the effects of either lesion alone and resulted in no apparent deficits.

## Highlights

• Reversal learning impaired after dorsomedial striatal dopamine depletion.
• No evidence of set-formation after excitotoxic subthalamic nucleus lesions.
• No impairments observed after both lesions together.

## Introduction

The subthalamic nucleus (STN) has been found to be an effective target for functional neurosurgical treatments designed to ameliorate the motor symptoms of Parkinson’s disease (PD) (Henderson and Dunnett, 1998, Bronstein et al., 2011). Surprisingly, however, relatively little is known about the contribution of the intact STN to motor control and cognitive functioning, and there remains a paucity of data concerning the neural and behavioral effects of lesions of the STN in combination with striatal dopamine (DA) depletion, as occurs in PD (but see Baunez et al., 1995, Phillips and Brown, 1999, Baunez and Lardeux, 2011).

Most of the research to date has focussed on the motor functions of the STN, perhaps because the motor symptoms of PD are the target for treatment by STN inactivation (Limousin et al., 1995). Motor behavior is also readily assessed in experimental animals. Data related to the *cognitive* sequelae of STN activation, both in isolation and in combination with striatal DA depletion, are conspicuously rare in the literature. Baunez and Robbins, 1997, Baunez and Robbins, 1999 addressed the issue of cognitive impairments following lesions of the STN, using the five-choice serial reaction time task (5CSRTT; see Carli et al., 1983), which includes measures of attention. They demonstrated multiple deficits, many of which required an explanation beyond a simple failure of motor inhibition, from which they concluded that the STN played an important role in attentional processing. Subsequent investigations of STN function in rats have maintained focus on high attentional-demand reaction time tasks, like the 5CSRTT, in lesion models (e.g. Chudasama et al., 2003), or in lower attentional-demand choice reaction time tasks after high-frequency stimulation (e.g. Darbaky et al., 2003, Desbonnet et al., 2004, Baunez et al., 2007, Li et al., 2010). STN deep brain stimulation (DBS) in PD patients has, however, been reported to improve (Daniele et al., 2003) or impair some forms of executive function (Saint-Cyr et al., 2000, Smeding et al., 2006), although data are inconsistent and thought to depend on pre-existing frontal dysfunction (see Fields and Troster, 2000, Bronstein et al., 2011), and to date there has been little exploration of executive function, beyond visuo-spatial attention, in rats.

In the present study, we examined the roles of the STN and dorsomedial striatum (DMS) DA in a well-established test of cognitive flexibility – the intradimensional/extradimensional (ID/ED) attentional set-shifting task – that has been adapted for rats (Birrell and Brown, 2000, Tait et al., 2014). The test, analogous to the Wisconsin Card Sorting Test (WCST) and formally the same as that used extensively in monkeys to explore the neural basis of attentional flexibility (e.g. Dias et al., 1996a, Dias et al., 1996b, Dias et al., 1997), involves acquisition of a series of two-choice discriminations based on responding to relevant perceptual features of complex stimuli, while ignoring other features that also distinguish the stimuli. Subsequent acquisition stages are then based either on the initial relevant perceptual feature (an ID acquisition), or attention must be shifted to a previously irrelevant feature (an ED shift acquisition). Reversals of the discriminations follow each acquisition stage. Crucially, this task relies on natural foraging behavior: it is self-paced, and does not require a high degree of motor coordination.

We were interested in the contributions of DMS DA and the STN, and their functional interactions, in the performance of this task. Data from human subjects with PD undertaking the ID/ED task show impaired ED shifting in both medicated and un-medicated patients (Downes et al., 1989, Owen et al., 1992) – with un-medicated patients specifically impaired at shifting to a previously irrelevant dimension (Owen et al., 1993). While impairments in reversal learning were not reported in these studies, PD patients do show impaired learning of probabilistic reversals both off (Peterson et al., 2009), and on (Cools et al., 2001, Shohamy et al., 2009) dopaminergic medication. Also, the ventral striatum is active in healthy humans during reversal learning (Cools et al., 2002a, Cools et al., 2004). The cognitive effects of striatal dysfunction have been well documented in rats. The reason we chose to lesion DMS, rather than dorsolateral striatum (DLS), is that previous evidence suggests that DMS is selectively implicated in reversal learning but not acquisition (Ragozzino et al., 2002a, Ragozzino et al., 2002b, O’Neill and Brown, 2007, Castane et al., 2010, Braun and Hauber, 2011), whereas lesions including DLS have been shown to result in greater impairments, including of acquisition (Featherstone and McDonald, 2004a, Featherstone and McDonald, 2004b) and motor-related (Kirik et al., 1998). While there is at least one report suggesting DMS is involved in stimulus–response acquisition (Featherstone and McDonald, 2005), in a two-choice bowl-discrimination foraging task, that was not the case (O’Neill and Brown, 2007), so by restricting the lesion to DMS, we hoped to see selective reversal learning impairments and avoid globally impaired performance. Therefore, rather than attempt to explore a model of PD per se, we have instead chosen to investigate a discrete region of the striatum, and cognitive, rather than purely motor, impairments associated with DA depletion in that area.

The first question of interest to us was whether large lesions centered on the STN would ameliorate specific cognitive deficits resulting from DMS DA depletion. As over-activity of the STN may be responsible for – and STN inactivation may alleviate – many of the motor symptoms of striatal DA depletion, STN inactivation might also be effective in ameliorating impaired cognitive functions, presumed to be subserved by parallel circuits through the basal ganglia (BG) (see Alexander and Crutcher, 1990, Mink and Thach, 1993). An ancillary question was whether large lesions of the STN might themselves result in cognitive impairments in this task and how the lesions might interact.

## Experimental procedures

### Animals

Thirty-two male Lister hooded rats (Charles River, UK) were housed in pairs in 25 × 45 × 15-cm plastic cages. Testing was conducted in the light phase of a 12-h light/dark cycle (lights on at 7:00 am). The rats were maintained on a restricted diet (15–20 g of food per day) with water freely available in the home cage. We adhered to the guidelines laid out in the Principles of Laboratory Animal Care (National Institutes of Health, Publication No. 86-23, revised 1985) and the requirements of the United Kingdom Animals (Scientific Procedures) Act 1986.

### Apparatus

The apparatus and task have been described previously (see Birrell and Brown, 2000, Tait et al., 2014). Briefly, rats were placed in a subdivided (three sections: one large holding area and two smaller ‘choice’ compartments) plastic cage and trained to dig in ceramic bowls to retrieve food reward (one half of a Honey Loop (Kellogg, Manchester, UK)). The bowls were placed in the smaller compartments, filled with a scented digging medium and with their outer surfaces and rims covered with a texture. Plexiglass panels were used to fully, or individually, occlude the choice chambers from the holding area.

### Habituation

On the day prior to testing, rats were placed in the apparatus for ∼60 min. Two sawdust-filled bowls were placed, one in each of the smaller compartments, with both containing reward. After a rat had obtained reward from both bowls, they were re-baited. When the rat was reliably digging, typically having obtained the reward from each bowl six times, it was trained on three two-choice simple discriminations (SD) – one for each dimension to be used during testing: texture, odor and digging medium. Trials were initiated by raising the divider to give the rat access to the two compartments, each containing a bowl discriminable by a different exemplar within a single dimension, with only one exemplar being rewarded. The rat had to dig for reward in the correct bowl on six consecutive trials to reach criterion. The first four trials were discovery trials: if the rat dug in the incorrect bowl, the trial was recorded as an error, but it was permitted to retrieve the reward from the correct bowl. On subsequent trials, if the rat dug incorrectly, an error was recorded, and access to the rewarded bowl was blocked. On all trials, the rat was permitted to continue digging in its chosen bowl until it had obtained the reward (correct trial) or moved away from the bowl (error trial), at which point access to both compartments was blocked to allow initiation of a new trial. The order, and correct/incorrect exemplars of the training SDs were identical for all rats: rubber vs masking tape (texture); blackcurrant vs vanilla tea (odor); or polystyrene pieces vs shredded paper (digging medium). Sawdust was the digging medium for both texture and odor SDs, and bowls with no added texture were used for odor and digging medium SDs.

### Testing

In a single test session, rats performed a series of two-choice discrimination stages: a novel SD; a compound discrimination (CD); a reversal (REV1); an ID; a second reversal (REV2); an ED shift; and a third reversal (REV3). At the CD, novel exemplars for one irrelevant dimension were added, but the correct exemplar from the SD remained correct. At the REV1 stage, the correct and incorrect exemplars from the CD were reversed. At the ID, novel exemplars from each dimension were introduced, with an exemplar from the previously relevant dimension being correct. At the REV2 stage, the reward status of the relevant ID exemplars was reversed. At the ED shift, novel exemplars from each dimension were introduced, with an exemplar from the previously irrelevant dimension being correct. At the REV3 stage, the reward status of the ED exemplars was reversed. The specific order that the exemplar pairs appeared in were not repeated within a group and were matched between groups. There were six possible directions of shift (odor to texture or medium, medium to odor or texture, texture to medium or odor), so each shift was used at least once in each group, and matched between groups as much as possible.

### Surgery

Anesthesia was induced with an intraperitoneal (i.p.) injection of pentobarbital sodium (1.0 ml/kg, 65 mg/ml) and all rats were pre-treated with an i.p. injection of the monoamine oxidase inhibitor, pargyline (50 mg/kg in warm sterile 0.9% saline; Sigma Chemical Co., Poole, UK) 30 min prior to surgery. Six rats received a bilateral injection of 0.4 μl of 0.06 M ibotenic acid (Tocris Cookson Ltd; Avonmouth, UK), in the STN at coordinates AP −3.8 mm; ML ±2.4 mm; DV −8.5 mm (from skull surface) with the tooth bar set to −3.3 mm to achieve level skull. Six rats received a bilateral injection of 8 μg 6-hydroxydopamine base (6-OHDA) in 2 μl of 0.01% ascorbate saline, at the coordinates AP +2.5 mm; ML ±1.8 mm; DV −4 mm (from skull surface) with tooth bar set at +5 mm. A further six rats received both the DMS 6-OHDA and the STN ibotenic acid injections, in the same surgery. Fourteen control animals received bilateral injections of saline in the striatum (*n* = 8) or STN (*n* = 6). All infusions were at a rate of 0.01 μl every 10 s with the cannula left *in situ* for a further three minutes. Testing was conducted between five and ten days after surgery. One rat from the DMS lesion group did not complete all stages of testing, and so this rat was excluded from the analysis.

### Histology

At the end of the experiment, rats were transcardially perfused with 4% paraformaldehyde in 0.1 M phosphate buffer after anesthesia with i.p. administered Euthatal® (1.0 ml/kg, pentobarbital sodium, 200 mg/ml). Brains were removed, placed into a 20% sucrose/4% paraformaldehyde solution, and stored at 4 °C overnight in a refrigerator. The following day, 50 μm coronal sections were cut using a freezing microtome for staining with cresyl violet or tyrosine hydroxylase immunoreactivity. Lesion damage was assessed by observing reduced tyrosine hydroxylase immunoreactivity in the striatum, or the extent of cell loss and gliosis in the STN.

### Data analysis

Trials to criterion and errors to criterion were recorded, however as the two measures are correlated and as analysis of either measure produced the same results, only the analysis of trials to criterion is reported. Repeated measures analysis of variance (ANOVA) was employed, with a within-subject factor of Stage (seven levels: SD, CD, REV1, ID, REV2, ED, and REV3) and a between-subject factor of Group (four levels: STN lesion, DMS lesion, combined lesion, and control). Restricted analyses with post-hoc comparisons were used to analyze significant interactions and test the source of main effects (Winer, 1971).

## Results

### Histology

**Fig. 1:**
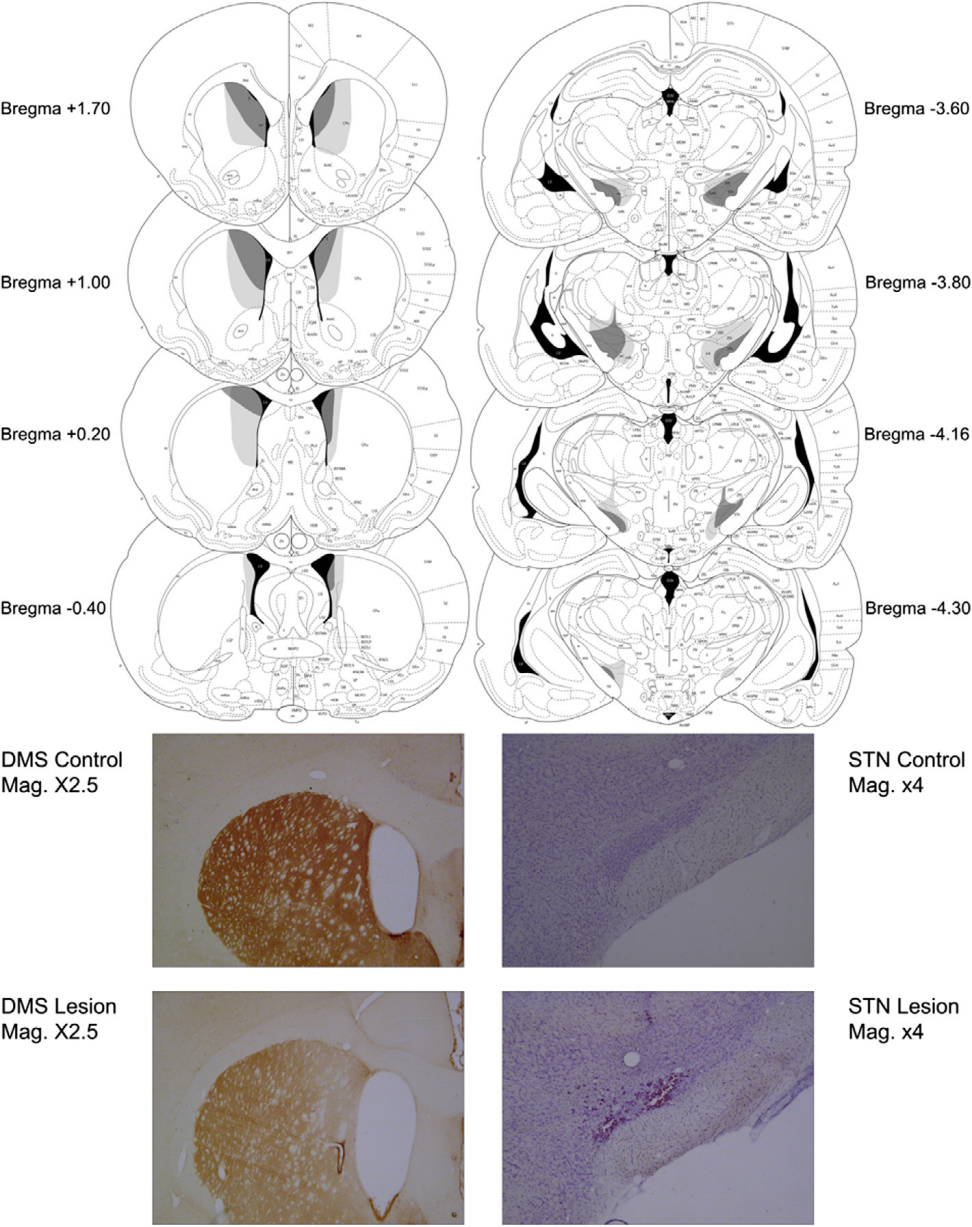
A series of coronal sections (adapted from Paxinos and Watson, 1998) and photomicrographs showing the striatum (right) and the subthalamic nucleus (left), to indicate the extent of typical small (dark shading) and large (pale shading) lesions. There was no systematic difference between the lesion groups: in particular, the lesions were neither more nor less extensive in the combined lesion group compared to the single lesion group.

Fig. 1 illustrates the extent of typical lesions on schematic sections, redrawn from the schematics of Paxinos and Watson (1998). Although there is no explicit boundary for the DMS region, reduced tyrosine hydroxylase was evident bilaterally in the dorsomedial portion of the striatum of all DMS-lesioned rats, although not equally extensively in all animals (the Fig. 1 schematic therefore shows typical small and large lesions – with the larger lesions presenting as greater spread of depletion rather than greater depletion in the same area as the small lesions). For the STN lesion group, lesions to the STN were almost complete, sparing only the most posterior sections, and including both the medial and lateral portions in all cases. Lesion damage extended into the zona incerta (ZI) to varying degrees, in all but one subject. Track damage was evident in the ventroposteromedial thalamus in five subjects and calcium deposits were evident in the entopeduncular nucleus in all cases.

### Behavioral results

**Fig. 2:**
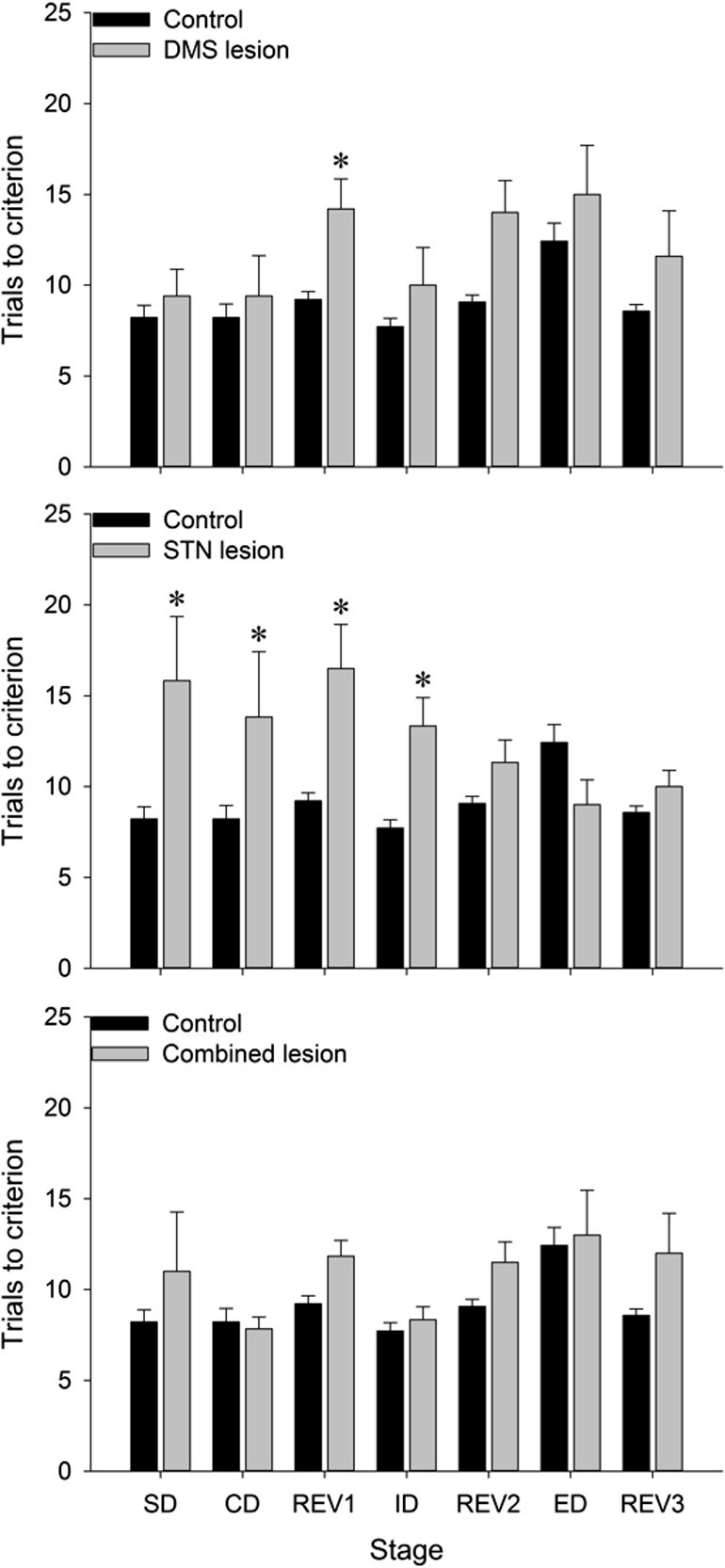
Bar graphs showing trials to a criterion (six consecutive correct trials) + SEM for each discrimination, in the order in which the discriminations were performed, for the three lesion groups (DMS lesion – top graph; STN lesion – middle graph; combined lesion – bottom graph). The data from the combined control group are repeated on all three graphs. ^*^p < 0.05.

Fig. 2 shows the trials to criterion for each of the discriminations, for each group of rats. As expected, there were differences between the different stages of the test (main effect of Stage, with Huynh–Feldt correction for a sphericity violation, *F*_6,162_ = 2.9, *p* < 0.05). Control rats learned a novel discrimination faster when it was based on the previously relevant perceptual dimension (ID stage) compared to the ED shift stage, when attentional set had to be shifted to the previously irrelevant dimension. Similarly, more trials were needed at the reversal stages than initial acquisition or the ID stage.

The different lesions affected performance overall during the task (main effect of Group, *F*_3,27_ = 5.2, *p* < 0.05: STN lesion vs control, *p* = 0.05; DMS lesion vs control, *p* = 0.05), and at different stages of the test (interaction between lesion Group and Stage, with Huynh–Feldt correction, *F*_18,162_ = 2.4, *p* < 0.05). The interaction was further analyzed using restricted ANOVA for each stage of the test (with F ratios corrected by using the mean square error term from the analysis of all of the data; see Winer, 1971) and uncorrected post-hoc comparisons to test the source of significant main effects.

#### Initial acquisition

At the SD stage, only the STN lesion resulted in more errors, with a mean increase of 7.6 trials compared to control (main effect of Group, *F*_3,27_ = 5.6, *p* < 0.05; STN lesion vs control, *p* < 0.05. None of the other groups differed from control). The STN group also made significantly more errors than the control group at the CD stage (main effect of Group, *F*_3,27_ = 3.4, *p* < 0.05).

#### Reversal learning

At REV1, rats with STN lesions and those with DMS lesions made significantly more errors than unlesioned controls (main effect of Group, *F*_3,27_ = 5.6, *p* < 0.05; STN lesion vs control, 7.3 additional trials, *p* < 0.05; DMS lesion vs control, 5.0 additional trials, *p* < 0.05). There was no significant difference between the combined lesion group and the controls (2.6 additional trials, *p* = 0.1). At the second and third reversal, there were no significant differences between the groups (main effect of group: *F*_3,27_ = 2.1 (REV2) and 1.4 (REV3), not significant (*ns*)).

#### ID and ED

At the ID stage, the STN lesion group made significantly more errors than the control group and the combined lesion group (main effect of Group, *F*_3,27_ = 5.9, *p* < 0.05: STN lesion vs control (+5.6 trials); STN lesion vs combined lesion (+5.0 trials), both *p* < 0.05). There was no difference between the STN lesion group and the DMS lesion group, likely due to the small overall increase in trials at all stages for the DMS lesion group, as there was no difference between control and DMS lesion ID performance.

Importantly, at the ED shift stage, there were no significant differences between the groups (main effect of Group, *F*_3,27_ = 2.3, *ns*) and there was no evidence of a selective impairment in any group in shifting of attentional set, which would be expected to result in an increase in the number of trials at the ED relative to the ID acquisition.

In terms of the cost of shifting set, none of the control rats took fewer trials to reach criterion at the ED shift stage than at the ID stage: the average difference between the ED and the ID for the controls was an additional 4.7 (standard error of the mean (SEM) 1.0) trials at the ED, which is regarded as indicative of an attentional set. Similarly, the rats with DMS lesions required an additional 5.0 (SEM 2.7) trials and the rats with combined lesions required an additional 4.7 (SEM 2.5) trials in the ED compared to the ID stage, suggesting the strength of attentional set and the ability to shift attention was the same in these groups. In the rats with STN lesions, however, the increased errors at the ID stage meant that all but one rat completed the ED in *fewer* trials than the ID, with the group mean being 4.3 (SEM 1.4) fewer.

## Discussion

Patients with PD are impaired in attentional set-shifting (Bowen et al., 1975, Owen et al., 1992, van Spaendonck et al., 1995, Gauntlett-Gilbert et al., 1999, Cools et al., 2002b), but we report here that discrete DMS DA-depleted rats, while experiencing a mild discrimination learning impairment, were not selectively impaired at ED shift discrimination learning. It also did not result in more errors for ID discrimination learning – i.e. the ED shift took more trials to solve than the preceding ID, suggesting that an attentional set had been formed and that shifting of set was normal. DMS DA depletion resulted in a reversal learning impairment, which is consistent with previous reports of DMS function after either cholinergic manipulation (Ragozzino, 2003, Tzavos et al., 2004, McCool et al., 2008), similar group-sized DMS-targeted DA depletion (O’Neill and Brown, 2007), and similar to the effects of quinolinic acid lesions to DMS (increased errors during reversal learning; Castane et al., 2010, Lindgren et al., 2013). Only performance at REV1 was significantly worse than in control rats, perhaps suggesting the reversal learning impairment is transient. Nevertheless, although not statistically significant, performance at both REV2 and REV3 was elevated compared to controls. It is worth noting that medial prefrontal cortex (mPFC) DA efflux increases during only the first of a series of lever-pressing reversals in rats (van der Meulen et al., 2007), so it may be the case that involvement of mPFC/striatal DA during initial reversal learning is limited to initial exposure to a reversal. However, a methamphetamine binge administration, which reduced DA transporters in the dorsal striatum (DS) of rats, impaired all three reversal stages in the ID/ED task (also with no effect on ID/ED performance; Izquierdo et al., 2010). We do not think that the lack of significant difference between DMS DA-depleted rats and control at REV2 and REV3 is sufficiently robust evidence for us to suggest that there is a genuine ‘recovery’ or even a significant improvement.

We have previously reported that orbital prefrontal cortex (PFC) lesions impair reversal learning in this task, which is persistent and has an associated set-formation impairment (McAlonan and Brown, 2003, Chase et al., 2012). We have also previously reported persistent reversal learning impairments, but with no effect on set-formation or shifting, in aging rats, which we attributed to changes in striatal cholinergic/DA interactions (Tait et al., 2013). With so few errors, it is difficult to determine the reasons for the present deficit (for example, whether there is increased perseveration or learned non-reward) without making specific manipulations to the task to test this (e.g. Tait and Brown, 2007). Nevertheless, DMS DA depletion-induced reversal learning impairments did not impact on attentional set-formation/shifting, implying the origin of the deficit is likely to be different to the orbital prefrontal effect. As we have also reported that DMS lesions result in reversal learning impairments after simple discriminations (SDs) (O’Neill and Brown, 2007), this, combined with a normal ID/ED relationship in the current data, would suggest that the compound nature of the discriminations was not a factor. This makes it unlikely that the reversal learning impairment seen here was due to the rat ‘opting out’ of the reversal by attending to other aspects of the stimuli, as has been suggested to account for reversal learning impairments in patients with PD (cf. Shohamy et al., 2009). Previous reports of perseverative responding in the 5CSRTT after striatal DA depletion (Baunez and Robbins, 1999) might suggest that perseveration is at the root of our observed reversal learning deficit. Nevertheless, it is particularly interesting to note that our STN/ZI lesions ameliorated this DMS lesion effect, whereas Baunez and Robbins (1999) reported that STN lesions exaggerated perseverative responding in 5CSRTT performance. It remains possible that these two forms of perseverative behavior reflect related, albeit distinct, cognitive processes, both of which are, nevertheless, mediated by the STN.

Although, as a group, the DMS DA-depleted rats showed a slight increase in the mean difference between the ED and ID stages (i.e., the shift-cost), this increase was not statistically significant. It is possible that this lack of impairment at the ED stage is due to a fundamental difference between species, although we regard this explanation as unlikely: the rat does form an attentional set and shifting set is impaired in the rat, as in primates, following lesions of the PFC (Birrell and Brown, 2000). Furthermore, we can rule out an explanation based on a difference between rodents and primates: in the marmoset, Crofts et al. (2001) also found no effect of striatal DA depletion at the ED stage of the analogous test. It is possible that the lesions in both the rat (present results) and marmoset (Crofts et al., 2001) were not extensive enough to impair set-shifting. Alternatively, it may be the case that DA-mediated attentional set-shifting deficits arise from interactions between PFC and striatum, rather than explicitly from DA dysfunction in the striatum alone.

Lesions of the STN/ZI area resulted in a quite different response profile. The rats required significantly more trials to learn the initial stages of the test (SD, CD, REV1, and ID), but between REV1 and the ED shift stage, they required progressively fewer trials. Furthermore, the ED stage in the STN/ZI-lesioned rats was not completed in more trials than their ID – i.e. the STN/ZI-lesioned rats showed no evidence of having formed an attentional set. The increased trials at the earlier stages of the test indicate that the rats were not discriminating optimally. Indeed, the behavior of the rats was noteworthy, with a tendency to start to dig in the first bowl approached, even when it was the incorrect bowl. Although response time was not recorded, it was apparent that the time spent digging in the incorrect bowl (i.e., the persistence of the digging) also decreased over trials within the stage, indicating that the rats were learning about which bowl was baited, but that their strategy for finding the bait was much less efficient. This behavior is possibly related to the increase in anticipatory responding in reaction time tasks, a consistent finding following bilateral (Baunez et al., 1995, Baunez and Robbins, 1997) and unilateral lesions of the STN (Phillips and Brown, 1999). Phillips and Brown (1999) noted that performance of STN-lesioned rats is sometimes normal once the response is under target control, but there is a failure to inhibit responses in the period preceding the target. Although, in the task used here, the stimuli are available to the rat (and therefore one might conclude that behavior was always under stimulus control), nevertheless the bowls must be explored sequentially. When the rat encounters the negative stimulus first, it is necessary to inhibit digging in that bowl and move to the other bowl. This inability to resist making a response could also be argued to be a form of perseveration – a previously rewarded response (digging) is repeated regardless of the outcome. As noted above, perseverative responding could take different forms and this persistent digging could be equivalent to the persistent nose-poking reported in the 5CSRTT (Baunez and Robbins, 1999). This perhaps suggests that the STN is implicated in lower order response-reward perseveration, rather than the higher order stimulus–response perseveration that would result in reversal learning impairments. STN lesions have been reported to impair ‘switching behavior’ (a form of reversal) in a visual cue-place discrimination in rats (Baker and Ragozzino, 2014), as well as stopping during a stop-signal reaction-time task (Eagle et al., 2008). STN neurons are also activated during switching between automatic and controlled eye saccades in monkeys (Isoda and Hikosaka, 2008). Each of these seemingly different behaviors could reflect a form of cognitive perseveration to a response. However, as with the DA depletion-induced reversal learning impairment, the STN/ZI lesion-induced persistent digging we observed was not present in the combined lesions. This does suggest that the perseveration observed in the 5CSRTT in STN/ZI-lesioned rats, which was enhanced in combined STN/ZI + striatal DA-depleted rats in the 5CSRTT (Baunez and Robbins, 1999), arises from a subtly different process than the effects reported here.

There remains the possibility of a role for the ZI in the behaviors we report. Our lesions extended into the ZI in the majority of animals, and the ZI is well-established as involved in both locomotor activity (e.g. Mogenson et al., 1985, Supko et al., 1991, Supko et al., 1992), and DA regulation in the BG in rats (Walker et al., 2010). In PD patients, the ZI is a target for DBS, and although it is not as effective at ameliorating motor deficits as STN-DBS, cognitive domains are relatively spared and WCST performance is not affected at all (Welter et al., 2014). Hershey et al. (2010). How this might correspond to the present results is difficult to conclude. The reported persistent digging may be a symptom of dysregulated motor control, brought slowly under cognitive control as the rats gain experience. Yet, as we cannot distinguish between the role of the STN and the ZI in our current cohort, it is equally possible that the persistent digging that we report is an effect of dysregulated motor control, cognition, or both. Thus, while we suggest that it is a transient lower order form of perseveration that underlies the impairment observed in the early stages of our task, it is clear that role of the STN and/or ZI in cognition is very much dependent on the specific task. Furthermore, more discrete lesions/manipulations of both regions are necessary to elucidate the roles of each.

Without an ID/ED difference in the STN/ZI-lesioned rats, it is difficult to draw conclusions about the effects of the lesion on attentional set – either formation or shifting. However, if the rats had formed an attentional set, but the benefit of being ‘on set’ at the ID was masked by persistent digging, it would be expected that by the point in the task at which the STN/ZI-lesioned rats were responding in a similar fashion to controls (REV2), the data should reflect the cognitive processes involved in discrimination learning and set-shifting, rather than the gradual improvement in withholding the persistent digging response. While there is no significant difference between ID and ED in the STN/ZI-lesioned rats, nor between lesion and control EDs, it is clear from the figure that even if the lesioned rats’ ID were at control levels, there would still be no difference between the ID and the ED. If attentional set had not formed, we would predict that ID would increase slightly (because no benefit would accrue from attending to the relevant dimension) and ED would decrease slightly (because there would be no cost of attending to the irrelevant dimension) such that performance between the two stages would be roughly equal. Thus, the low number of trials to criterion in the lesioned rats’ ED, regardless of their performance at the ID stage, suggests that there was no ‘cost’ to shift attentional set. The most likely explanation for this is that the acquisition of an attentional set was, for whatever reason, compromised.

Intriguingly, the rats with combined lesions (both DMS DA depletion and excitotoxic STN/ZI lesions) showed neither pattern of impairment. As a group, their performance did not differ significantly from controls at any stage of testing. Two rats had patterns of errors that resembled those seen in the groups with single lesions (i.e., for one, a large number of errors at the SD stage while, for the other, there were a large number of errors on the reversal stages) even though in both cases the STN/ZI lesion was complete and the DA depletion from the DMS were qualitatively similar to that of the other rats. The pattern of errors of the remaining four rats with combined lesions was within the range of the control group.

These data are particularly significant given the effect of STN lesions on reaction time performance of rats with striatal DA depletion – rats with combined lesions perform exactly like rats with lesions of the STN alone, with normal reaction times but an increase in anticipatory errors (Baunez et al., 1995, Baunez and Robbins, 1999, Phillips and Brown, 1999). Several authors – including ourselves – have concluded that STN lesions appear to improve deficits resulting from striatal DA depletion while resulting in additional deficits (e.g. Baunez et al., 1995, Henderson and Dunnett, 1998, Baunez and Robbins, 1999, Phillips and Brown, 1999). In this study, we have shown that the additional deficits arising from STN/ZI lesions alone are not necessarily seen when combined with DMS DA depletion. This is potentially a particularly important finding because the effects of lesions of the STN are typically interpreted in the context of its position ‘down-stream’ from the striatum, thus subject to disruption – in particular, over-activity – as a result of striatal DA depletion (see Wichmann and DeLong, 1996); or in its position in an alternative pathway from the cortex through the BG (e.g. the ‘hyper-direct’ pathway; Nambu, 2004) and thus independent of striatal output. The present data suggest that just as STN/ZI lesions may ameliorate the effects of DMS DA depletion, the interaction of the STN and the striatum is possibly more complex than this, as DMS DA depletion here appeared to ameliorate the effects of STN/ZI lesions – a phenomenon not previously observed.

In patients with PD, Daniele et al. (2003) reported beneficial effects of STN DBS on a version of the WCST. However, there have also been reports of no change (Funkiewiez et al., 2004, Heo et al., 2008), short-term (absent by six months post-operative) impairments (Aono et al., 2014), and longer term left hemisphere STN DBS-induced impairments (Lueken et al., 2008) on WCST performance. To our knowledge, there are no published data on ID/ED tasks after STN DBS in human patients with PD, although there are consistent reports of decline in verbal fluency resulting from STN DBS (e.g. Gironell et al., 2003, Morrison et al., 2004, Parsons et al., 2006). Early reviews (Kalteis et al., 2002, Woods et al., 2002, Bronstein et al., 2011) concluded that any positive cognitive effects of STN DBS are not as obvious as the positive motor effects, with more recent assessments (Combs et al., 2015, Da Cunha et al., 2015) suggesting, in addition to reduced verbal fluency, there is an inconsistent trend for mild impairments in mood and other cognitive domains (e.g. visuomotor processing speed; Follett et al., 2010, but see Odekerken et al., 2013). Nevertheless, the present data suggest that destruction of the STN can result in improvements in non-motor deficits arising from DMS DA depletion.

## Conclusion

We have shown that performance in a self-paced task, with low demands on motor competence, which measures the acquisition and shifting of attentional set, is impaired following DMS DA depletion and STN/ZI lesions, but in different ways. The impairment following DMS DA depletion is during reversal learning while STN/ZI lesions resulted in a distinctive pattern of responding, characterized by persistent digging, with abnormal learning and possibly a failure to form an attentional set. Both deficits are fully remediated in rats with combined DMS and STN/ZI lesions.
